# Retrospective Evaluation of a Minor Dietary Change in Non-Diabetic Group-Housed Long-Tailed Macaques (*Macaca fascicularis*)

**DOI:** 10.3390/ani11092749

**Published:** 2021-09-20

**Authors:** Dian G. M. Zijlmans, Annemiek Maaskant, Elisabeth H. M. Sterck, Jan A. M. Langermans

**Affiliations:** 1 Animal Science Department, Biomedical Primate Research Centre, 2288 GJ Rijswijk, The Netherlands; maaskant@bprc.nl (A.M.); e.h.m.sterck@uu.nl (E.H.M.S.); langermans@bprc.nl (J.A.M.L.); 2 Animal Behaviour and Cognition, Department of Biology, Utrecht University, 3508 TB Utrecht, The Netherlands; 3 Unit Animals in Science & Society, Department Population Health Sciences, Faculty of Veterinary Medicine, Utrecht University, 3584 CM Utrecht, The Netherlands

**Keywords:** adiposity, feed, fiber, health, non-human primate, nutrition

## Abstract

**Simple Summary:**

Macaques in captivity are prone to becoming overweight and obese, which may cause several health and welfare problems. Diet likely plays an important role herein. In an attempt to reduce overweight incidence and related health problems, a minor dietary change was implemented in our long-tailed macaque breeding colony. The provisioning of bread was replaced by grains and vegetables, while the basic diet of monkey chow remained the same. Overweight status did not differ after dietary change, but some biochemical parameters related to glycemic response and lipid metabolism improved. This study emphasizes the importance of evaluating husbandry changes and shows that relatively minor dietary adjustments may improve animal health and welfare.

**Abstract:**

Macaques in captivity are prone to becoming overweight and obese, which may cause several health problems. A diet that mimics the natural diet of macaques may prevent these problems and improve animal welfare. Adjusting captive diets towards a more natural composition may include increasing fiber content and lowering the glycemic index, i.e., reducing the impact on blood glucose levels. Such a dietary change was implemented in our long-tailed macaque (*Macaca fascicularis*) breeding colony. The basic diet of monkey chow pellets remained the same, while the supplementary provisioning of bread was replaced by grains and vegetables. This study is a retrospective evaluation, based on electronic health records, that investigated whether this minor dietary change had a beneficial effect on relative adiposity and overweight-related health parameters in 44 non-diabetic, group-housed, female long-tailed macaques. Relative adiposity was measured with a weight-for-height index and blood samples were collected during yearly health checks. Glycemic response and lipid metabolism were evaluated using several biochemical parameters. Relative adiposity and overweight status did not differ after dietary change. Yet, relatively heavy individuals generally lost body weight, while relatively lean individuals gained body weight, leading to a more balanced body weight dynamic. Dietary change did not affect HbA1c and triglyceride levels, while fructosamine and cholesterol levels were significantly reduced. Thus, the minor dietary change had no significant effect on overweight status, but some biochemical parameters related to the risk of diabetes and cardiovascular disease were positively affected. This study emphasizes the importance of evaluating husbandry changes and that critically reviewing husbandry practices can provide valuable insights to improve animal health and welfare.

## 1. Introduction

Macaques in captivity are susceptible to becoming overweight and obese. Similar to humans, this can cause several health problems, such as type 2 diabetes mellitus (T2DM) and cardiovascular disease [1,2]. Diet likely plays an important role in becoming overweight and the related health problems [3]. A diet that mimics the natural diet of macaques may prevent these problems. Wild macaques mainly eat wild fruits, supplemented with seeds, flowers, leaves, buds, bark and small animals, e.g., insects [4,5,6,7]. This natural diet is high in fiber and low in fat [8,9], resulting in little to no overweight in wild macaques [10,11]. In contrast, diets in captivity tend to be low in fiber and high in easily digestible carbohydrates, such as sugar [12]. Accordingly, 10–15% of captive macaques develop obesity during their life [13].

Adjusting the diet towards a more natural composition may decrease overweight-related health problems and improve animal welfare in captivity. For example, increasing fiber and decreasing sugar content in the diet led to a reversal of prediabetes and more natural behaviour in great apes [12]. Fruits, vegetables, and grains generally contain a high amount of fiber and have a low glycemic index (GI ≤ 55) [14,15,16]. Other food items, such as bread, have a high GI (GI ≥ 70) as they contain carbohydrates that are quickly digested and metabolized [14,17]. This leads to postprandial hyperglycemia, i.e., a high increase in blood glucose after consumption, which has been proposed to increase the risk of T2DM and cardiovascular disease in humans [16].

Various biochemical parameters can be used to assess the risk of developing overweight-related health problems such as TD2M and cardiovascular disease. As animals are progressing towards T2DM, the glycemic response becomes impaired and blood glucose levels increase [18]. As a result, glycated proteins are formed, e.g., fructosamine and glycated hemoglobin (HbA1c), both accurate biomarkers to measure the intermediate and long-term glycemic response, respectively [19,20]. In addition, obese macaques experience changes in markers for lipid metabolism, e.g., increased total cholesterol and triglyceride concentrations, which are risk factors for the development of both T2DM and cardiovascular disease [18,21]. These four biochemical parameters provide information regarding health risks and can thus be useful in the diagnosis and management of overweight-related health problems in macaques [20].

In an attempt to reduce overweight incidence and overweight-related health problems, a dietary change was implemented in our long-tailed macaque (*Macaca fascicularis*) breeding colony. The supplementary provisioning of bread was replaced by grains and vegetables, while the basic diet of monkey chow pellets remained the same. Although wild long-tailed macaques mainly eat fruits, it would not be appropriate to feed similar amounts of fruit in captivity. Cultivated fruits have a different nutritional composition, i.e., less protein and fiber and more sugar, compared to wild fruits [12,22]. Since the nutritional composition of cultivated vegetables is more like wild fruits, more vegetables than fruits were provided. The dietary change led to an increased fiber content and a lower GI. The implementation of this dietary change had little impact on daily husbandry practices, i.e., feeding times and routines remained unchanged.

This study is a retrospective evaluation that investigated whether this minor dietary change had a beneficial effect on relative adiposity and overweight-related health parameters in non-diabetic, group-housed, female long-tailed macaques. The evaluation was based on data retrieved from electronic health records. Relative adiposity and biochemical parameters were measured during annual health checks before and after dietary change. Overweight status was determined with a species-specific weight-for-height index, which represents relative adiposity levels of long-tailed macaques [11]. Biochemical parameters related to glycemic response, i.e., fructosamine and HbA1c, and lipid metabolism, i.e., cholesterol and triglyceride, were compared to evaluate the effect of dietary change on the risk of T2DM and cardiovascular disease.

## 2. Materials and Methods

### 2.1. Subjects and Housing

Subjects of this study were 44 full-grown adult female long-tailed macaques from the breeding colony of the Biomedical Primate Research Centre (BPRC), an AAALAC accredited facility, in Rijswijk, the Netherlands. The animals were aged between 6 and 22 (10.7 ± 0.61) years old and weighed between 3.4 and 9.15 (5.5 ± 0.20) kg at the time of initial data collection. Pregnant and (pre)diabetic individuals were excluded to prevent the possibility of pregnancy or disease progression interfering with our outcome parameters. All females lived with their offspring and typically with one adult breeding male in multi-generational groups (N = 9 groups). The groups were formed by adhering to natural group dynamics, i.e., females are philopatric, while males are removed from their natal group at puberty. The amount of data on adult males was therefore insufficient to include them in the data analyses.

Individuals had access to enriched indoor (±72 m^2^ and 2.85 m high) and outdoor (±250 m^2^ and 3.1 m high) compartments. The inside enclosure contained sawdust bedding, while the outside enclosure had a sand bedding where natural plant growth was possible. Environmental enrichment consisted of several climbing structures, beams, fire hoses, car tires, sitting platforms, and a swimming pool to stimulate natural behaviour [23]. Drinking water was freely available throughout the day via automatic water dispensers.

### 2.2. Diet and Dietary Change

The basic diet of the macaques consisted of monkey chow pellets (Ssniff, Soest, Germany) that were daily fed in the morning. The amount of monkey chow per individual was calculated based on the basal metabolic rate and depended on their age, sex, and body weight [24]. Adult females were calculated to require on average 90 g of monkey chow per day. In addition, one slice of wheat bread (~30 g, three times a week), 120 g of fruit/vegetables (three times a week), or 15 g of a grain mixture (once a week) were provided per individual in the afternoon (Table 1). Since the sum of all individuals’ needs was provided to the group and the distribution of food among group members could not be controlled, actual food intake likely varied per individual. Food enrichment was provided occasionally but its contribution to daily nutritional intake was carefully controlled and considered negligible.

A dietary change took place in June 2019 to reduce the diet’s glycemic index and enhance fiber content. The supplementary provisioning of wheat bread in the afternoon (three times a week) was replaced by maize silage, grain mixture, and vegetables. As a result, the ratio of fruit to vegetables changed from approximately 1:3 before dietary change to 1:5 after dietary change. The 10 most commonly fed fruit/vegetables were banana, bell pepper, cabbage, chicory, Chinese cabbage, cucumber, endive, leek, lettuce, and tomato (Table A1). The dietary change led to a 15.4% increase in fiber content in the average daily diet, while the amount of energy, protein, carbohydrates, and fat remained approximately the same (Table 2). Besides an increase in fiber content, the removal of bread led to a lower overall GI after dietary change.

### 2.3. Data Collection

This retrospective evaluation was based on data retrieved from BPRC’s electronic health records, which included data from annual health checks. These health checks are a routine veterinary procedure related to the regular health management of the colony [25]. No additional procedures were performed, and all procedures complied with regulations in the European Directive 2010/63 and the Dutch law. The health checks prior to dietary change took place in spring 2018. Since the dietary change took place in June 2019, data from the health checks in autumn 2020 were used for testing the effect of the dietary change. Subjects served as their own control to exclude possible confounding factors, e.g., dominance rank, genetics, etc.

Prior to the health checks, individuals were fasted overnight, while water was freely available throughout the night. At the assessment in spring 2018 (before dietary change), individuals were sedated with an intramuscular injection of ketamine (10 mg/kg, Ketamine 10%; Alfasan, Woerden, The Netherlands). There was a subsequent change in the routine anesthesia protocol for the benefit of the animals. In autumn 2020 (after dietary change), monkeys were thus sedated with a combination of ketamine (10 mg/kg, Ketamine 10%; Alfasan, Woerden, The Netherlands) and medetomidine (0.05 mg/kg, Sedastart; AST Farma, Oudewater, The Netherlands) IM, which was reversed after the procedures with atipamezole (0.25 mg/kg, Sedastop; AST Farma, Oudewater, The Netherlands) administration IM. Medetomidine induces muscle relaxation and results in mild hyperglycemia [26,27,28].

As part of the health check, body weight and height were determined, as described earlier [11]. Briefly, a standard scale was used to measure body weight to the nearest 0.1 kg. Height was measured as crown–rump length by placing the monkeys on their back on a measuring mat (SECA, Hamburg, Germany). Height was measured to the nearest 0.1 cm. Body weight and height were used to calculate a species-specific weight-for-height index (hereafter referred to as WHI). WHI was calculated as weight (in kilograms) divided by height (in meters) to the power of 2.7 (WHI2.7 in [11]). This measure of relative adiposity was preferred over solely using body weight as the latter does not take into account individual variation in height. Although all females were full-grown and skeletally matured, height was highly variable (range: 40.1–47.7 cm). We determined overweight status and individuals were considered overweight when their WHI exceeded the upper boundary of 62 kg/m^2.7^ [11].

Furthermore, blood samples were collected for complete blood count and blood chemistry. The samples were analyzed for fructosamine (umol/L), HbA1c (%), total cholesterol (mmol/L) and triglyceride (mmol/L) levels using a Cobas Integra 400 plus (Roche Diagnostics, Rotkreuz, Switzerland). Blood samples were collected from the vena femoralis into EDTA and serum tubes (Vacuette, Greiner Bio-One international GmbH, Alphen aan den Rijn, The Netherlands), left for 30 min and centrifuged at 3000 rpm for 10 min. Afterwards, the remaining serum was transferred to polypropylene tubes and stored below −20 °C.

All biochemical parameters, except for triglyceride after dietary change, were analyzed on the same day of the sample collection. Triglyceride levels after dietary change were analyzed roughly five months after sample collection. A correction was applied to the data as triglyceride levels in serum are only stable up until three months when stored at −20 °C [29]. The correction was based on the regression equation between triglyceride levels after five months and deviation from the actual value from thirty samples for which the original triglyceride values were available (Appendix B).

### 2.4. Data Analyses

Statistical testing was performed in IBM SPSS Statistics version 26. The effect of dietary change on body weight, WHI, and biochemical parameters was tested with a paired samples *t*-test or Wilcoxon signed ranks test. Normal distribution of the data was checked with the Shapiro–Wilk test. Pearson and Spearman correlations were used to test the association between age and WHI and associations between the different biochemical parameters. Linear regression analyses were used to evaluate whether age and WHI affected delta WHI, fructosamine, HbA1c, cholesterol, and triglyceride levels. Normality and homoscedasticity of the residuals were visually checked. Delta WHI was calculated as WHI after dietary change (in 2020) minus WHI before dietary change (in 2018). Finally, Fisher’s exact test was used to compare the proportion of overweight individuals before and after dietary change. Descriptive statistics in the results are reported as mean ± SE. The level of significance was α = 0.05 and all tests were two-tailed.

## 3. Results

### 3.1. Relative Adiposity and Overweight Status

**Figure 1:**
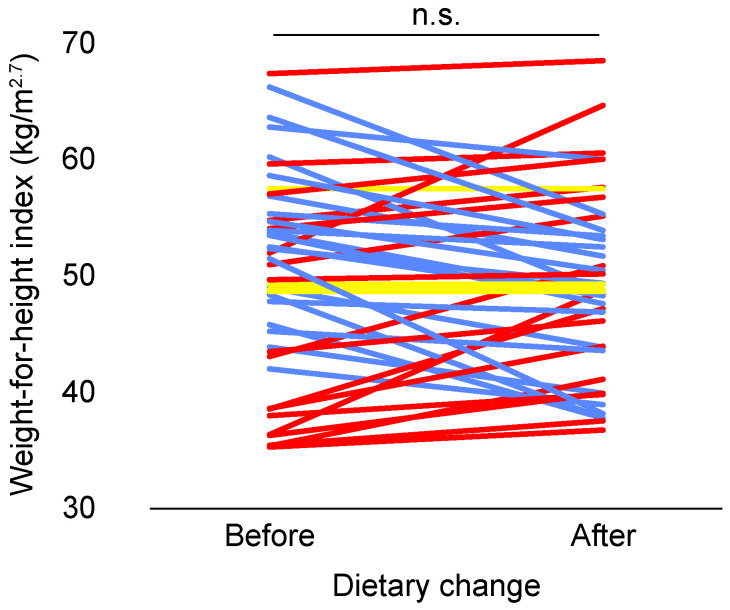
Effect of dietary change on weight-for-height index (WHI) in adult female long-tailed macaques. Each line represents an individual (*n* = 44). Blue lines represent a decrease, while red lines indicate an increase and yellow lines represent no change. n.s. means non-significant (*p* ≥ 0.05).

**Figure 2:**
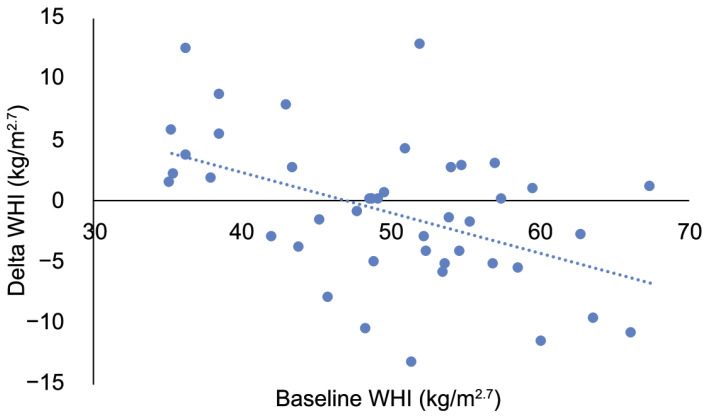
Delta weight-for-height index (WHI) plotted against the baseline WHI in adult female long-tailed macaques that were subjected to a minor dietary change.

Mean body weight was 5.5 ± 0.20 kg before dietary change and 5.4 ± 0.19 kg after dietary change, which is not a statistically significant difference (paired samples *t*-test, *t* = 0.959, *n* = 44, *p* = 0.343). Similarly, WHI did not differ after dietary change (paired samples *t*-test, *t* = 0.991, *n* = 44, *p* = 0.327; Figure 1). WHI was independent of age in our study population both before (Spearman correlation, *r* = 0.180, *n* = 44, *p* = 0.242) and after dietary change (Spearman correlation, *r* = 0.053, *n* = 44, *p* = 0.731). Delta WHI was independent of age (F (1, 41) = 0.689, *p* = 0.411), but was significantly associated with baseline WHI (F (1, 41) = 11.731, *p* = 0.001). Delta WHI was significantly higher in individuals with a low baseline WHI, implying that WHI increased in relatively lean individuals, while WHI decreased in relatively heavy individuals after dietary change (Figure 2).

The year before dietary change, four individuals (9.1%) had WHIs above the upper boundary for overweight, while only two individuals (4.5%) were overweight after dietary change. Overweight status did not significantly differ before or after dietary change though (Fisher’s exact test, *p* = 0.676).

### 3.2. Biochemical Parameters

Table 3 shows descriptive statistics on fructosamine, HbA1c, cholesterol, and triglyceride levels before and after dietary change. Correlations between the different biochemical parameters were weak or absent (Table A2).

#### 3.2.1. Glycemic Response

**Figure 3:**
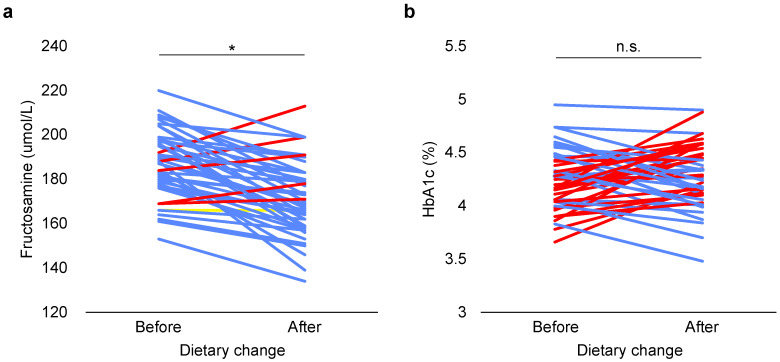
Effect of dietary change on fructosamine (**a**) and HbA1c levels (**b**) in adult female long-tailed macaques. Each line represents an individual (*n* = 44). Blue lines represent a decrease, while red lines indicate an increase and yellow lines represent no change. * means statistically significant difference, *p* < 0.05; n.s. means non-significant (*p* ≥ 0.05).

Fructosamine levels were independent of age and WHI both before (F (1, 41) = 0.053, *p* = 0.820; F (1, 41) = 1.520, *p* = 0.225) and after dietary change (F (1, 41) = 1.698, *p* = 0.200; F (1, 41) = 0.026, *p* = 0.873). After the dietary change, fructosamine levels were significantly reduced (paired samples *t*-test, *t* = 7.060, *n* = 44, *p* < 0.0005; Figure 3a).

WHI had no significant influence on HbA1c levels before (F (1, 41) = 0.057, *p* = 0.812) or after dietary change (F (1, 41) = 1.214, *p* = 0.277). Age did not affect HbA1c levels before dietary change (F (1, 41) = 0.680, *p* = 0.414), yet age was positively associated with HbA1c levels after dietary change (F (1, 41) = 7.261, *p* = 0.010), i.e., older individuals had higher HbA1c levels. The regression equation indicated that HbA1c values increased 0.028% per year of age (R^2^ = 0.174). Dietary change had no significant effect on HbA1c levels (paired samples *t*-test, *t* = −0.759, *n* = 44, *p* = 0.452; Figure 3b).

#### 3.2.2. Lipid Metabolism

**Figure 4:**
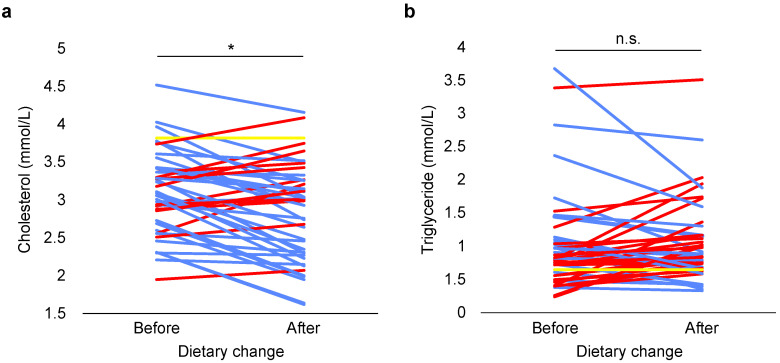
Effect of dietary change on cholesterol (**a**) and triglyceride levels (**b**) in adult female long-tailed macaques. Each line represents an individual (*n* = 44). Blue lines represent a decrease, while red lines indicate an increase and yellow lines represent no change. * means statistically significant difference, *p* < 0.05; n.s. means non-significant (*p* ≥ 0.05).

Cholesterol levels were independent of age and WHI before (F (1, 41) = 0.164, *p* = 0.687; F (1, 41) = 0.257, *p* = 0.615) and after dietary change (F (1, 41) = 0.082, *p* = 0.775; F (1, 41) = 2.567, *p* = 0.117). Cholesterol levels were significantly reduced after dietary change (paired samples *t*-test, *t* = 3.971, *n* = 44, *p* < 0.0005; Figure 4a).

Triglyceride levels were independent of WHI before (F (1, 41) = 0.793, *p* = 0.378) and after dietary change (F (1, 41) = 3.353, *p* = 0.074), while triglyceride levels significantly increased with age before (F (1, 41) = 7.146, *p* = 0.011) and after dietary change (F (1, 41) = 5.491, *p* = 0.024). The regression equations showed that triglyceride levels increased with every additional year of age with 0.072 mmol/L before dietary change (R^2^ = 0.177) and 0.051 mmol/L after dietary change (R^2^ = 0.181). Triglyceride levels were not significantly different after dietary change (Wilcoxon signed ranks test, Z = −0.604, *n* = 44, *p* = 0.546; Figure 4b).

## 4. Discussion

This study evaluated, based on electronic health records, the effect of a minor dietary change on relative adiposity and overweight-related health parameters in non-diabetic, group-housed, female long-tailed macaques. Relative adiposity and biochemical parameters related to glycemic response and lipid metabolism were compared before and after the supplementary provisioning of bread was replaced by grains and vegetables. Relative adiposity and overweight status did not differ after dietary change. Yet, relatively heavy individuals generally lost body weight, while relatively lean individuals gained body weight, leading to a more balanced body weight dynamic. Dietary change had no effect on HbA1c and triglyceride levels, while fructosamine and cholesterol levels were significantly reduced. Thus, the minor dietary change had no significant effect on overweight status but had a positive effect on some biochemical parameters related to the risk of T2DM and cardiovascular disease.

### 4.1. Relative Adiposity and Overweight Status

Body weight, WHI, and overweight status did not differ after dietary change. Based on the increased fiber content, a reduction in relative adiposity was expected. Fiber intake increases satiety and decreases the feeling of hunger after a meal, which results in reduced energy intake, even when food is available ad libitum [30]. The importance of fiber in the diet of captive primates is increasingly being recognized and the provisioning of browse (e.g., willow twigs), which is high in fiber, is therefore often recommended and becoming more popular in zoos and other institutions [31,32]. A reduction in body weight after transitioning to a high-fiber diet was found in a vervet monkey (*Chlorocebus aethiops sabaeus*) breeding colony [33], but not in our study. However, the relative increase in fiber content was almost tenfold higher in the vervet monkey study (140%) compared to our study (15.4%). Higher fiber contents may be needed for relative adiposity to decrease overall.

Nevertheless, dietary change had a differential effect on relative adiposity of individual animals, depending on their baseline value. Relatively lean individuals gained body weight, while relatively heavy animals generally lost body weight after dietary change. This finding may be explained by an unexpected secondary effect of the dietary change. Although food intake was not measured in this study, this finding suggests that dietary change resulted in a different distribution of food among group members. Wheat bread was easy to monopolize, resulting in some individuals obtaining several slices, while others obtained none (personal observation, cf. [34,35]). In contrast, grains, maize silage, and leafy vegetables were likely divided more equally, as these items were spread through and/or in front of the cages. The more equal distribution of these food items may have led to relatively lean individuals obtaining more food than before, thus gaining body weight, while relatively heavy individuals obtained less food, thereby losing body weight. Even though relative adiposity did not decrease overall, dietary change had a differential effect on individual animals resulting in a more balanced body weight dynamic.

Relative adiposity was not related to any of the biochemical parameters in this study, while other studies found several associations between being overweight and indicators of glycemic response and lipid metabolism in macaques. Cholesterol and triglyceride levels are generally higher in obese male and female rhesus macaques (*Macaca mulatta*) compared to their non-obese counterparts [1,21,36]. Body weight is also positively correlated with triglyceride and glucose levels in adult female long-tailed macaques [37]. These studies often included highly obese subjects with body fat accounting for up to 61% of their body weight [1]. This body fat percentage would equal a body weight of roughly 12.65 kg [38], while the heaviest monkey in our study initially weighed 9.15 kg. Accordingly, no effect of body weight on cholesterol, triglyceride, or glucose levels is found in long-tailed macaques with relatively low body weights [39]. Thus, the relatively low overweight prevalence and little variation in relative adiposity between individuals in our study may explain the lack of significant associations between WHI and biochemical parameters.

The absence of these relationships implies that this long-tailed macaque population is generally healthy regarding overweight-related health parameters. All biochemical parameters, i.e., fructosamine, HbA1c, cholesterol, and triglyceride levels, also fit well within previously reported ranges for this species [18,19,39,40,41,42,43,44]. However, this does not mean that overweight-related health problems do not occur in our breeding colony.

### 4.2. Glycemic Response

Since a high-GI food (bread) was replaced with low/medium-GI foods (grains and vegetables), the glycemic response was expected to improve, thereby reducing the risk of T2DM. Glycemic response was measured using fructosamine and HbA1c levels, which produced different results regarding the effect of dietary change. Fructosamine levels decreased, while HbA1c levels showed no significant difference after dietary change. These results may be explained by the difference in sensitivity of albumin and hemoglobin to bind to glucose. Fructosamine is formed when plasma glucose binds to albumin, while HbA1c results from glycation of hemoglobin [20]. Albumin has been suggested as being more sensitive to postprandial glycemic variation compared to hemoglobin and therefore larger alterations in blood glucose would be needed to affect HbA1c levels similar to fructosamine levels [45,46]. As a result, varying fiber and glucose intake in humans does not affect HbA1c, but significantly influenced fructosamine levels [46]. Similarly, fructosamine levels differ between long-tailed macaques fed a standard or high-fat diet, while no difference is found in HbA1c [19]. These findings are consistent with the outcome of our study. Although no significant effect on HbA1c levels was found, the decrease in fructosamine suggests that the dietary change had a positive impact on glycemic response. As the decrease in fructosamine was observed across the study population, this was likely a primary effect of dietary change and independent of the potentially new food distribution.

A third possible biochemical parameter to quantify glycemic response is plasma glucose concentration. Although plasma glucose levels were measured, a fair comparison was not possible as medetomidine is known to affect glucose levels and this was added to the anesthesia protocol [27,28]. Moreover, plasma glucose levels provide information about instant glucose levels, while fructosamine levels reflect blood glucose levels from the past two to three weeks and HbA1c represents the previous two to three months [20]. Fructosamine and HbA1c are thus more suitable parameters to detect long-term changes in glycemic response as they reflect glucose levels over a longer period.

Nevertheless, there was no significant association between fructosamine and HbA1c levels. Cefalu et al. (1993) found that fructosamine and HbA1c are significantly correlated (*r* = 0.61) in a long-tailed macaque population, which included both diabetic and non-diabetic monkeys [19]. Fructosamine and HbA1c also correlate well in diabetic humans (0.55 < *r* < 0.88; [47,48,49,50]), but no correlation has been found in non-diabetic humans (*r* = 0.01; [50]). Our study included only non-diabetic individuals, which might explain the lack of correlation between fructosamine and HbA1c.

Furthermore, HbA1c levels increased with age after dietary change, but this was not found before dietary change. HbA1c levels are also positively associated with age in non-diabetic humans [51,52,53], but not in other studies with macaques and squirrel monkeys (*Saimiri* species; [44,54]). Since higher age is a risk factor for the development of T2DM in both humans and primates [18,55], the link between age and HbA1c and their relation to T2DM in primates may need further investigation.

### 4.3. Lipid Metabolism

Multiple studies show that an increased fiber intake has a positive effect on lipid metabolism, i.e., leads to reduced total cholesterol, LDL and triglyceride levels, in humans and rats [56,57]. Especially water-soluble fibers seem to have this cholesterol-lowering effect in humans [58]. Therefore, it was expected that the serum cholesterol and triglyceride levels would decrease after the dietary change. In line with this expectation, cholesterol levels decreased after dietary change. In contrast, triglyceride levels showed no significant difference after dietary change.

In the present study, higher triglyceride levels were found in older individuals compared to younger monkeys both before and after the dietary change. Similar age-effects have been reported in other studies with both long-tailed macaques and rhesus macaques [42,43,59,60]. Possibly, no significant effect of dietary change on triglyceride levels was found because the effect of dietary change was counteracted by an age-effect. Furthermore, triglyceride levels after dietary change had to be corrected due to the period between blood sample collection and analysis. This correction may have introduced some bias, thereby reducing reliability of the triglyceride data.

Altogether, the minor dietary change had a beneficial effect on at least one of the two biochemical parameters related to cardiovascular disease.

## 5. Conclusions

This study evaluated in retrospect the effect of a minor dietary change on relative adiposity and overweight-related health parameters in non-diabetic, group-housed, female long-tailed macaques. The basic diet of monkey chow pellets remained the same, while the supplementary provisioning of bread was replaced by grains and vegetables. Although this minor dietary change had no significant effect on overweight status, dietary change had a differential effect on individual animals resulting in a more balanced body weight dynamic. Also, some biochemical parameters related to the risk of diabetes and cardiovascular disease were positively affected. These results emphasize the importance of evaluating husbandry changes and shows that critically reviewing husbandry practices can provide valuable insights to improve animal health and welfare.

## Figures and Tables

**Table 1:** Nutritional values of food items in the daily diet of female long-tailed macaques at BPRC based on the (average) amount fed per animal. Information was obtained from the food item’s manufacturer or The Dutch Food Composition Database (NEVO, online version 2019/6.0; Table A1).

Food Item	Amount (gram)	Energy (kcal)	Protein (gram)	Carbs (gram)	Fiber (gram)	Fat (gram)	Glycemic Index [14]
Monkey chow	90	295	22.7	32.5	3.78	3.87	NA #
Fruit/vegetables	120	33	1.48	4.97	2.40	0.31	Low
Maize silage	120	71	3.12	17.6	7.01	1.38	Low
Grain mixture	15	50	1.66	10.7	0.47	0.38	Medium
Wheat bread	30	59	2.20	11.8	1.34	0.40	High

^#^ NA = not available.

**Table 2:** Average daily intake of energy, protein, carbohydrates, fiber, and fat before and after dietary change in adult female long-tailed macaques.

Nutritional Component	Before Dietary Change	After Dietary Change	Relative Change (%)
Energy (kcal)	342	339	−1.0%
Protein (gram)	24.5	24.4	−0.2%
Carbohydrates (gram)	41.2	40.9	−0.7%
Fiber (gram)	5.45	6.29	+15.4%
Fat (gram)	4.23	4.35	+3.0%

**Table 3:** Descriptive statistics on biochemical parameters related to glycemic response and lipid metabolism before and after dietary change. *n* = 44. Mean ± SE (minimum–maximum) are reported. * *p* < 0.05.

	Before Dietary Change	After Dietary Change
Fructosamine (umol/L)	187 ± 2.3 (153–220)	171 ± 2.5 (134–213) *
HbA1c (%)	4.25 ± 0.04 (3.66–4.95)	4.29 ± 0.04 (3.48–4.90)
Cholesterol (mmol/L)	3.10 ± 0.08 (1.95–4.52)	2.84 ± 0.10 (1.62–4.16) *
Triglyceride (mmol/L)	1.02 ± 0.11 (0.24–3.68)	1.05 ± 0.09 (0.33–3.51)

**Figure A1:**
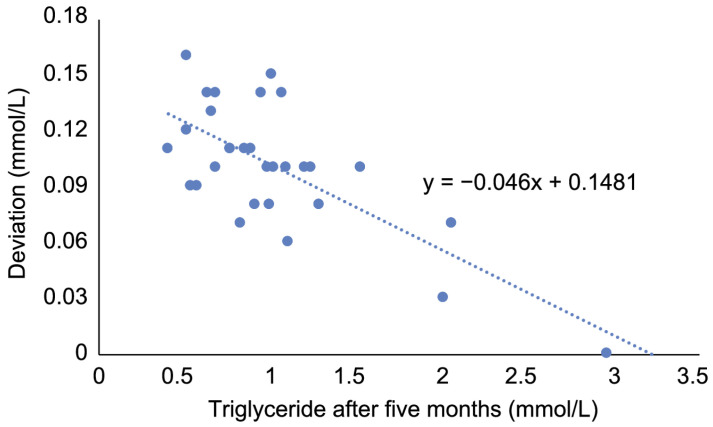
Deviation in triglyceride levels plotted against the triglyceride levels when analyzed after being stored at −20 °C for five months (*n* = 30).

## Data Availability

Data are available on reasonable request.

## Appendix A

**Table A1:** Nutritional values of the 10 most commonly fed fruit and vegetables at BPRC (in alphabetical order) based on the amount fed per adult: 120 g. Source: The Dutch Food Composition Database (NEVO, online version 2019/6.0).

Fruit/Vegetable	Energy (kcal)	Protein (gram)	Carbohydrates (gram)	Fiber (gram)	Fat (gram)
Banana	110	1.32	24.0	2.28	0.36
Bell pepper	30	0.84	4.68	3.00	0.24
Cabbage	43	2.88	4.92	4.92	0.24
Chicory	23	1.56	2.88	1.44	0.24
Chinese cabbage	20	1.20	2.40	3.00	0.00
Cucumber	16	0.84	1.56	0.72	0.48
Endive	19	1.80	1.20	2.04	0.36
Leek	34	1.80	4.20	3.60	0.24
Lettuce	16	1.68	0.36	1.44	0.48
Tomato	24	0.84	3.48	1.56	0.48

**Table A2:** Correlations between different biochemical parameters in adult female long-tailed macaques (*n* = 44). The type of correlation, correlation coefficient (*r*), and significance (*p*) are reported ^1^.

	Fructosamine (umol/L)	HbA1c (%)	Cholesterol (mmol/L)	Triglyceride (mmol/L)
Fructosamine (umol/L)	X	Pearson, *r* = 0.025, *p* = 0.873	Pearson, *r* = 0.229, *p* = 0.135	Spearman, *r* = 0.205, *p* = 0.182
HbA1c (%)	Pearson, *r* = 0.233, *p* = 0.127	X	Pearson, *r* = -0.370, *p* = 0.014	Spearman, *r* = 0.071, *p* = 0.646
Cholesterol (mmol/L)	Pearson, *r* = -0.223, *p* = 0.145	Pearson, *r* = 0.035, *p* = 0.822	X	Spearman, *r* = −0.010, *p* = 0.950
Triglyceride (mmol/L)	Spearman, *r* = 0.212, *p* = 0.167	Spearman, *r* = 0.220, *p* = 0.151	Spearman, *r* = −0.012, *p* = 0.940	X

^1^ The numbers in the lower left triangle represent correlations before dietary change, while the upper right triangle contains the correlations after dietary change.

## Appendix B

Triglyceride samples were analyzed roughly five months after blood collection, while triglyceride is only stable for three months when stored at −20 °C [29]. To check the reliability of the triglyceride levels analyzed after five months, we used 30 samples for which the original triglyceride values (i.e., analyzed on the same day as blood collection) were available. These samples were re-analyzed after being stored at −20 °C for five months. We then calculated the deviation between triglyceride levels analyzed on the same day as blood collection and triglyceride levels analyzed five months after blood collection. All values after five months appeared to be equal to or higher than the original value. The deviation depended on the actual triglyceride level with deviation being higher in samples with lower triglyceride levels (Figure A1). The data resulted in the following regression equation: y = −0.046x + 0.1481, with y being the deviation and x being the triglyceride concentration after five months. This equation was used to correct the data.
